# Brain Injury Awareness Month — March 2020

**DOI:** 10.15585/mmwr.mm6909a1

**Published:** 2020-03-06

Brain Injury Awareness Month, recognized each March, provides an important opportunity to bring attention to the prevention of traumatic brain injury (TBI) and to promote strategies to improve the quality of life for persons living with TBI and their families.

TBIs, caused by an impact or force to the head or body or a penetrating injury to the head, affect millions of U.S. persons each year (*1*). Falls are a leading mechanism of TBI, and older adults are at increased risk for sustaining a TBI and experiencing TBI-associated adverse outcomes (*1*,*2*). A report in this issue of *MMWR* found a nation­wide 17% increase in the rate of fall-related TBI deaths during 2008–2017, with increases in most states (*3*). The largest increases in fall-related TBI deaths occurred among persons aged ≥75 years.

Evidence-based prevention efforts to decrease falls are important to reducing the incidence and prevalence of TBI among older adults. CDC’s STEADI (Stopping Elderly Accidents, Deaths & Injuries; https://www.cdc.gov/steadi/index.html) initiative includes resources and tools for health care providers to improve identification of patients at risk for a fall, as well as effective strategies to reduce the risk for fall-related injuries, including TBI.
